# The Incidence of Liver Damage Found during Postmortem Examination at the Slaughterhouse

**DOI:** 10.3390/ani13050839

**Published:** 2023-02-25

**Authors:** Lenka Valkova, Eva Voslarova, Simona Nincakova, Annamaria Passantino, Vladimir Vecerek

**Affiliations:** 1 Department of Animal Protection and Welfare and Veterinary Public Health, Faculty of Veterinary Hygiene and Ecology, University of Veterinary Sciences Brno, 612 42 Brno, Czech Republic; 2 Central Veterinary Administration of the State Veterinary Administration, 120 00 Prague, Czech Republic; 3 Department of Veterinary Sciences, University of Messina, Polo Universitario Annunziata, 981 68 Messina, Italy

**Keywords:** livestock, poultry, liver, veterinary inspection, pathological findings

## Abstract

**Simple Summary:**

The liver is the principal organ involved in animal metabolism. A healthy liver is critical to proper protein, carbohydrate, and fat metabolism. In contrast, liver damage results in a deterioration of animal health and, thereby, their well-being. In this study, liver damage was monitored in all animals reared on Czech farms and slaughtered in slaughterhouses in the Czech Republic for a period of 12 years (2010 to 2021). The results show the highest prevalence of liver damage in cattle, followed by pigs, sheep, and then goats, both in adults and in fattening animals. Chronic lesions were significantly more frequent than acute or parasitic lesions in the majority of species and categories of animal species, with the exception of piglets and ducks, in which acute lesions were most frequent, and ewes and lambs, in which parasitic lesions were most frequent.

**Abstract:**

We monitored liver damage in cattle (cows, heifers, fattening bulls, and calves culled from the herd), pigs (sows, finishing pigs, and piglets culled from the farm), sheep (ewes and lambs), goats (does and kids), rabbits, and poultry (end-of-lay hens, broiler chickens, turkeys, domestic ducks, and domestic geese) in the period from 2010 to 2021. All animals (*n* = 1,425,710,143) reared on Czech farms and slaughtered at slaughterhouses in the Czech Republic were included in the analysis. We determined the total number of damaged livers for individual categories of animals and also analyzed separately the incidence of damage of acute, chronic, parasitic, and other origin. The overall incidence of liver damage was higher in adult animals compared to fattening animals in all species. In cattle and pigs, the incidence was also higher in young animals culled from the herd compared to fattening animals. When comparing adult animals by species, the incidence of liver damage was highest in cows (46.38%), followed by sows (17.51%), ewes (12.97%), and does (4.26%). When comparing fattening animals by species, the incidence was highest in heifers (14.17%) and fattening bulls (7.97 %), followed by finishing pigs (11.26%), lambs (4.73%), and kids (0.59%). When comparing young culled from the herd by species, it was higher in piglets (32.39%) than in calves (17.6 %), and when poultry and rabbits were compared, the incidence was highest in turkeys (3.38%), followed by ducks (2.20%), geese (1.09%), broiler chickens (0.08%), and rabbits (0.04%). The results indicate that fattening animals have a better liver condition than mature animals and that culled young have a worse liver condition than older fattening animals. Chronic lesions represented the dominant proportion of pathological findings. Parasitic lesions occurred, first and foremost, in animals grazed on meadows with likely parasitic invasion, i.e., in ewes (7.51%), lambs (3.51%), and heifers (1.31%), and in animals in which antiparasitic protection is limited in view of the protection of meat from antiparasitic residues, i.e., finishing pigs (3.68%). Parasitic damage to the liver was rarely detected in rabbits and poultry. The results obtained represent a body of knowledge for measures to improve the health and condition of the liver in food animals.

## 1. Introduction

The liver is the principal organ involved in the metabolism of fat, carbohydrate, protein, vitamins, and minerals and in the immunity of animals [1], constituting their lifeline system. In contrast, liver damage results in a deterioration of animal health and, thereby, their well-being. A good understanding of all the factors that affect the function of the liver is important in view of the fact that the liver is the core of many digestive and metabolic processes. In cases of dietary imbalance or too-rapid feed transitions, the liver can be considerably affected and it could lead to the accumulation of waste and toxins. The influence of individual aspects of nutrition (the nutritional composition of the feed ration, feed restriction, anti-nutritional factors, the structural composition of the feed, nutritional supplements, etc.) on the size, development, and function of the liver must be thoroughly investigated. Research has shown that the composition of the prepartum diet also has an impact on lipid metabolism, liver function, and the overall health of the cow postpartum [2,3]. Petit et al. [4] have demonstrated that feeding saturated fatty acids increased the risk of fatty liver, while feeding with sources of unsaturated fatty acids, e.g., flaxseed (3.3% and 11.0% of the dry matter in prepartum and postpartum diets, respectively) during the transition period could increase liver concentrations of glycogen and decrease liver triglycerides after calving. A recent study has demonstrated that sheep can develop hepatic steatosis due not only to negative but also to positive energy balance based on excess carbohydrate energy [5]. Conversely, in swine, although they develop metabolic-syndrome-like features on a high-fructose diet, a substantial dietary fat is required to develop hepatic steatosis [6]. The weight of the liver appears to increase or decrease proportionally to the nutritional plan [7] and across physiological stages [8]. It has been reported that in cattle and sheep, an increase in the functional workload of the liver through dietary manipulation resulted in an increase in the liver weight associated with hepatocyte hypertrophy [8], unlike as observed in rats, in which a decrease in liver metabolism due to the surgical manipulation caused hepatocyte hypertrophy [9]. Attention must also be focused on feed-processing technology (such as heat treatment, pelleting, particle size, and the modification of whole-grain products) in terms of its impact on the state of the liver tissue [10]. For example, the use of amoniation–fermentation (amofer) technology for a complete feed formula has shown a significant benefit on livestock, such as feed efficiency, feed conversion, body weight increment, animal productivity, and liver function [11,12]. Liver damage generally occurs when the metabolism is disrupted, most often due to deficiencies in animal nutrition, or when the liver is damaged as a result of parasitic invasion as a target organ or an organ of the migration of parasites as part of the standard or random migration path in the animal organism. The liver may also be damaged as a result of an infectious process [13] or by intoxication of the organism (by heavy metals or mould toxins, for example) [14,15]. In addition, a housing system can predispose animals to certain lesions. For example, fatty liver syndromes are mainly found in caged hens, as reported by Keutgen et al. [16], who investigated pathologic changes in laying hens in various housing systems. 

The results of an extensive Lithuanian study [17] show that pathological lesions are detected most frequently in the respiratory system of livestock animals (specifically, in 35.61% of all slaughtered animals), closely followed by lesions on the liver (recorded in 34.19% of all slaughtered animals), during postmortem veterinary examination at Lithuanian slaughterhouses. Pathological findings in the livers of slaughtered animals are mentioned in a number of studies that investigated the reasons for the condemnation of livestock carcasses during postmortem veterinary examination. The majority of these studies have, however, focused only on a specific species, e.g., cattle [18,19,20], pigs [21,22,23,24,25], rabbits [26,27,28,29], broiler chickens [30,31,32], laying hens [16], and turkeys [33].

The aim of this study was to compare the incidence of liver damage in different species and categories of animals slaughtered at slaughterhouses. This study also aimed to distinguish the incidence of damage of acute, chronic, and parasitic origin.

## 2. Materials and Methods

Liver damage was monitored in animals slaughtered at slaughterhouses as part of the veterinary supervision of the health safety of the organs of slaughtered animals performed during post-slaughter veterinary inspection as required by the relevant European Union legislation [34]. Data for analysis were obtained retrospectively from the information system of the State Veterinary Administration of the Czech Republic, which contains information on the number of slaughtered animals and the results of veterinary inspections recorded by official veterinary inspectors of the Regional Veterinary Administrations of the State Veterinary Administration operating at slaughterhouses in the Czech Republic. For the purposes of the study, data on all animals reared on Czech farms and slaughtered for human consumption at any slaughterhouse in the Czech Republic between 2010 and 2021 were collected. A total of 1,425,710,143 animals were included in the analysis, namely 1,348,393 cows, 315,406 heifers, 1,214,298 fattening bulls, 120,238 calves, 683,912 sows, 29,628,524 finishing pigs, 152,088 piglets, 26,026 ewes, 132,553 lambs, 1680 does, 7690 kids, 2,403,779 rabbits, 23,116,811 end-of-lay hens, 1,328,693,065 broiler chickens, 1,516,365 turkeys, 52,360 geese, and 36,296,955 ducks. The animals were transported to the slaughterhouse in accordance with the requirements of the Council Regulation (EC) No 1/2005 [35] and slaughtered in accordance with the requirements of the Council Regulation (EC) No 1099/2009 [36].

The veterinary examination of slaughtered animals was conducted by official veterinarians who distinguished between livers without damage and livers with damage and recorded the nature of any liver damage found, distinguishing between acute damage, chronic damage, damage of parasitic origin, and other damage (usually icteric changes). The classification of findings was based on the methodology for postmortem inspection and was performed in a uniform way by slaughterhouse veterinary inspectors, i.e., specialized official veterinarians who received a specific training and obtained a certificate of competence.

Pathological changes caused by an inflammatory process lasting a short period before slaughter were classified as acute damage. These changes included, e.g., marked hyperemia, liver fragility, the presence of hemorrhages, swelling, increased organ size, and liver abscesses. 

Pathological changes caused by an inflammatory process lasting for a prolonged period before slaughter were classed as chronic damage. These changes included changes to the original structure of the tissue parenchyma involving penetration of the connective tissue, the formation of connective tissue scarring, and the appearance of adhesions. The chronic damage was also indicated by a reduction in the size of the liver and its stiffness, lighter color, smooth or wrinkled surface, the presence of cysts or calcifying abscesses, etc. A specific case of chronic changes is fatty degeneration manifested by enlargement of the liver, with the edges of the liver rounded and the liver being fragile and having a yellowish-brown color.

Changes that pointed to the invasion and migration of parasites and pathological processes caused by parasites in the host organism were classed as parasitic damage. Parasitic changes were not included in acute or chronic changes but were recorded separately in order to determine the extent of parasitic invasions. Parasitic damage included changes typical of parasitic invasion of the liver, i.e., focal changes characterized by small white spots or already-white scars on the surface of the liver (e.g., ascariasis in pigs—findings of scars in the liver after invasion of ascarids; coccidiosis in rabbits—findings of scars in the liver after invasion of coccidia), or directly with the finding of parasites in the bile ducts or in the liver tissue (e.g., *Fasciola magna*, *Fasciola hepatica*, etc.).

The incidence of liver damage was monitored in cattle, namely in cows (females after the first calving), heifers (females from 6 months of age to the first calving), fattening bulls (males over 6 months of age), and calves (up to 6 months of age, culled from the herd prematurely); in pigs, namely in sows (females after the first farrowing), finishing pigs (pigs that have reached the market weight), and piglets (from 10 weeks to premature slaughter (before reaching market weight); in sheep, namely in ewes (mature females) and lambs (around 4–6 months after reaching market weight); in goats, namely in does (mature females) and kids (around 3–6 months after reaching market weight); in rabbits; and in poultry, namely in end-of-lay hens, broiler chickens, turkeys, ducks, and geese.

The number of slaughtered animals and the number of damaged livers broken down into damage of chronic, acute, parasitic, and other origin were determined for each category of slaughtered animal over the entire monitored period of 12 years. The incidence of liver damage was expressed as a percentage.

The overall incidence of liver damage as well as the incidence of chronic, acute, and parasitic lesions were compared between individual species and categories of slaughtered animals. Furthermore, the incidence of liver damage was compared between fattening animals (fattening bulls, heifers, finishing pigs, lambs, kids), adult animals (cows, sows, ewes, does) and young animals culled from the herd for inadequate condition or health (calves, piglets).

The results were evaluated statistically using the program Unistat 6.5 for Excel. For the purposes of statistical comparison of the frequency of pathological findings in individual categories, a chi-square test was used to assess the statistical significance in a 2 × 2 contingency table [37]. Yates’ correction was used on frequencies exceeding 5, while Fisher’s exact test was used at frequencies lower than 5. Spearman’s rank test was used to assess the trend in the number of pathological findings [37]. The result of testing was the determination of Spearman’s rank correlation coefficients (rSp) used to assess a positive or negative trend in the numbers of findings. A value of *p* < 0.05 was considered statistically significant.

## 3. Results

**Figure 1:**
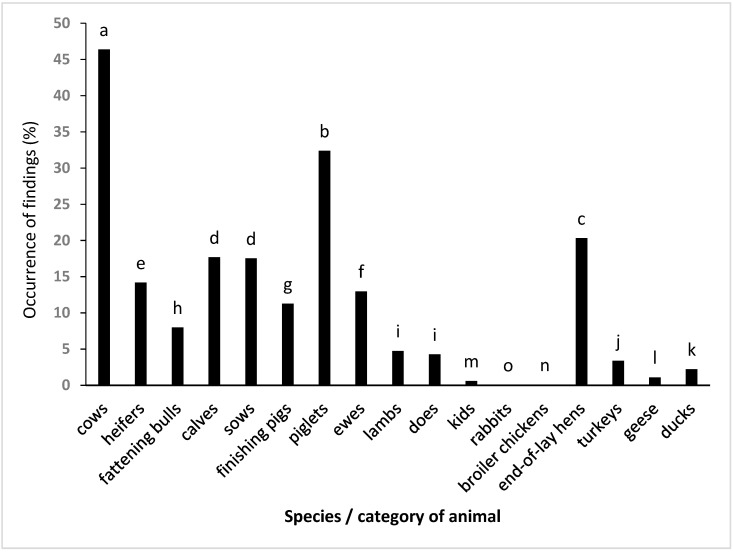
The overall occurrence of liver damage in individual categories of slaughtered animals. ^a–o^ values with different letters are significantly different at *p* ˂ 0.05.

The incidence of liver damage in individual categories of slaughtered animals is shown in Figure 1. The statistical comparison revealed significant (*p* < 0.05) differences between individual species and categories, with the exception of the comparison of goats and lambs (*p* = 0.10) and sows and calves (*p* = 0.36).

The results show that the incidence of liver damage was in all species higher in adult animals compared to fattening animals. It was higher in cattle and pigs in young culled from the herd compared to fattening animals. When individual species of adult animals were compared, it was highest in cows, followed by sows, sheep, and goats; when individual species of fattening animals were compared it was highest in heifers and fattening bulls, followed by finishing pigs, lambs, and kids; and when poultry and rabbits were compared it was highest in turkeys, followed by ducks, geese, broiler chickens, and rabbits.

The numbers of slaughtered animals and the occurrence of liver damage broken down into acute, chronic, parasitic, and other changes are shown in Table 1. Chronic changes were found to be more frequent (*p* < 0.05) than acute or parasitic changes in most species and categories of animals, with the exception of piglets and ducks, in which the most frequent changes were acute changes, and of sheep and lambs, in which the most frequent changes were parasitic changes. The occurrence of chronic and acute liver damage shows a similar structure to that of the overall changes. In contrast, the incidence of liver damage of parasitic origin was higher in adult animals compared to fattening animals only in sheep and goats, with the opposite being true in cattle and pigs. In cattle and pigs, the incidence of parasitic damage as higher in fattening animals in comparison with young animals culled from the herd. When adult animals were compared by species, the incidence of parasitic damage was highest in ewes, followed by does, cows, and sows, and when animals for fattening were compared by species, it was highest in finishing pigs, followed by lambs, heifers, bulls, and kids. Parasitic changes were found to be rare in poultry and rabbits.

The results of the Spearman rank correlations of the 12-year data within an animal category showed a significantly decreasing trend over the years for heifers, fattening bulls, ewes, lambs, kids, and end-of-lay hens and a significantly increasing trend over the years for calves, piglets, rabbits, and turkeys (Table 2).

## 4. Discussion

The occurrence of liver damage is significantly higher in adult animals compared to fattening animals. This is the case in cattle (cows 46.38% vs. fattening bulls 7.97% and heifers 14.17%), pigs (sows 17.51% vs. finishing pigs 11.26%), sheep (ewes 12.97% vs. lambs 4.73%), goats (does 4.26% vs. kids 0.59%), and domestic chicken (end-of-lay hens 20.32% vs. broiler chickens 0.08%). This finding is evidence of the fact that fattening animals are organisms with better liver health than adult animals. The burden on the liver is shorter-lasting in young animals in view of their age, and they also have better regenerative capacity, which is manifested in a lower number of abnormalities in the liver. With respect to the liver, young animals are for this reason in better condition than adult animals whose livers are burdened by long-term intensive metabolism resulting from long-term intensive production. Diets that are high in grain may increase liver abscesses in fed cattle [38]; however, this was not the case in animals monitored in our study. Whereas feedlot cattle in North America are typically fed high-grain diets (a ration is usually 70 to 90% grain and protein concentrates), in the Czech Republic, fattening bulls are typically fed a maize-silage-based diet containing only 30 to 35% of grain.

Dairy cows, in particular, though also suckler cows, remain on farms significantly longer than other categories of livestock animals. The average length of the productive life of dairy cows on farms ranges between 4.5 and 6 years. This period may be up to twice as long on average in the case of suckler cows [39]. In dairy cows in particular, a long rearing period connected with intensive milk production has an effect on the state of health and on the possible occurrence of production diseases that are associated with characteristic findings in the organs, including the liver. Chronic liver lesions are common in old cows [40]. Consequently, Dupuy et al. [40] documented the influence of age on the rate of condemnations at slaughterhouses and found the highest condemnation rate in animals slaughtered at the age of 5–10 years (37.37%).

As far as farmed species of birds are concerned, a relatively long lifespan (compared to broiler chickens or fattening turkeys, for example) combined with intensive egg production is typical of laying hens in particular. The rearing period, the housing system (cage housing still continues to prevail in the Czech Republic), and the use of laying hens are significantly different from other slaughtered poultry. While broiler chickens, geese, ducks, and turkeys are slaughtered after a few weeks or months of fattening (and are therefore usually young birds in good condition), laying hens are sent to slaughter after around a year of intensive egg production. In addition, it has also been shown that cage housing (compared to housing on litter or free-range housing) is one of the factors that contribute to the development of fatty liver syndrome in laying hens [16]. Our study confirmed these assumptions, with liver lesions recorded in 20.32% of all slaughtered laying hens, which is the highest proportion by far among poultry and the third-highest overall (after cows and piglets). The probable cause of the unsatisfactory condition of the liver in end-of-lay hens is nutrition that does not correspond to the intensity of their production (250–320 eggs per laying period, average egg weight 60–63 g). Laying hens may experience liver and muscle proteolysis, adipose tissue lipolysis, and bone resorption if they do not receive sufficient nutrients relative to the level of egg production [41]. The effect of age and the method and period of rearing is particularly evident in the difference between the number of liver lesions in end-of-lay hens (chronic 17.76%, acute 2.56%) and broiler chickens (chronic 0.04%, acute 0.03%), i.e., different categories within the same species of animal (the domestic chicken).

The results of this study also show that the incidence of liver damage is significantly higher in young culled from the herd (calves, piglets) compared to fattening animals (fattening bulls, heifers, finishing pigs). This is the case in cattle (calves 17.67% vs. fattening bulls 7.97% and heifers 14.17%) and in pigs (piglets 32.39% vs. finishing pigs 11.26%). This finding is evidence of the fact that young animals culled from the herd due to poor condition or poor health also have a lower level of liver health than animals kept in the herd, i.e., fattening animals (even when they are older than the culled young).

When adult cattle, pigs, sheep, and goats are compared, the incidence of liver damage is highest in cows (46.38%), followed by sows (17.51%), ewes (12.97%), and does (4.26%). This finding is evidence of the fact that, from the perspective of the liver, the greatest burden is seen in cows, probably as a result of the greatest difference between nutrients provided in the diet and metabolic needs, which leads to an enormous burden on the liver resulting in chronic or acute changes to the liver tissue. During early lactation, high-yielding dairy cows often develop a severe negative energy balance due to insufficient feed intake. Consequently, fat depots are mobilized in order to provide NEFA (non-esterified fatty acids) as an energy fuel. As a consequence of fat mobilization, the hepatic fat stores increase in the postpartum period. Excessive NEFA concentrations and a high liver fat content can lead to metabolic imbalances (i.e., ketosis and fatty liver syndrome) [42,43]. The effect of productivity on the condemnation rate at slaughterhouses in Switzerland was mentioned by Vial et al. [44], with this being considerably higher in dairy cattle (79%) compared to beef cattle (21%). Januskeviciene et al. [17] and Ceccarelli et al. [45], who did not differentiate between individual categories of cattle in their research, reported pathological findings (detected during postmortem veterinary examination) in cattle livers in 25.09% and 7.87% of all the examined animals, respectively. Ceccarelli et al. [45] reported distomatosis, followed by liver abscesses and hydatidosis (*Echinococcus granulosus*), as the commonest findings in the livers of cattle.

The impact on the liver is significantly lower in sows, likely due to the nature of nutrition based on feed mixtures with better control of nutrients in the diet. Nevertheless, pigs are a species of livestock animal in which a relatively large number of liver abnormalities were recorded in our study (piglets 32.39%, sows 17.51%, finishing pigs 11.26%). A high rate of condemnation in pigs due to liver lesions (9.85%, 31.79%, and 17.20%, respectively) was also reported by Ceccarelli et al. [45], Januskeviciene et al. [17], and Vecerek et al. [23]. Januskeviciene et al. [17] and Ceccarelli et al. [45], in agreement with our study, found the largest number of pathological lesions in the livers of cattle and pigs, though in contrast to our study they recorded a relatively larger number of lesions in pigs than in cattle.

The impact on the liver is even lower in sheep whose nutrition is provided by grazing in the vegetation season, when the organism regulates the supply of nutrients with regard to its metabolism by choosing its feed itself. In goats, nutrition is provided in stalls and, to a limited extent, also by grazing, and special attention is devoted to goat nutrition with a view to their general sensitivity to the level of nutrition and changes in nutrition, which is reflected in a low number of pathological changes in the liver. The generally better state of health of sheep and goats compared to cattle and pigs is confirmed by the studies by Januskeviciene et al. [17] and Ceccarelli et al. [45], who detected pathological lesions in the livers of 2.87% of all slaughtered sheep and goats, which corresponds quantitatively to our findings. According to the findings reported by Ceccarelli et al. [45], the liver was the organ most frequently affected pathologically in sheep (the liver accounted for 77.15% of all condemned organs in sheep). Sheep are reared largely under extensive rearing conditions in the Czech Republic. Sheep are reared all year round or for most of the year on pasture, which may be associated with a risk of the occurrence of parasites [46]. Unlike sheep, goats are not typical grazing animals and it is natural for them to feed selectively primarily on the leaves of bushes and trees. Unlike sheep, they also avoid soiled sites [47,48]. The risk of infection by parasitic life cycle stages may therefore be lower in goats, which may also be a reason for the better level of liver health seen in goats compared to sheep [49].

The occurrence of liver damage significantly differs also when individual species of fattening animals are compared, ranking in the order cattle, pigs, sheep, and goats, i.e., heifers (14.17%) and fattening bulls (7.97%), finishing pigs (11.26%), lambs (4.73%), and kids (0.59%). The same order of species in fattening animals as in adult animals is also evidence of a similar disproportion between the actual nutrition and metabolic needs in fattening animals and in adult animals.

The occurrence of liver damage also significantly differs when individual species of fattening poultry and rabbits are compared. The highest incidence is in turkeys (3.38%), followed by ducks (2.20%), geese (1.09%), broiler chickens (0.08%), and rabbits (0.04%). The results corroborate the higher sensitivity of turkeys to the correct content of nutrients in the diet, while in broiler chickens and rabbits, the low incidence of liver damage is evidence of the appropriate composition of feed mixtures with a view to the metabolic needs of broiler chickens and rabbits at the individual stages of their fattening.

The rabbits reaching the slaughterhouse are animals in the category of fattening rabbits, which means that they are individuals in good condition. Liver lesions were not among the frequent reasons for the condemnation of the carcasses or organs of rabbits at Polish slaughterhouses [28]. Hepatic coccidiosis, for example, was detected in 0.015% of all slaughtered rabbits during postmortem veterinary examinations performed in the period from 2010 to 2018 [28]. In the years between 2007 and 2011, meanwhile, hepatic coccidiosis was detected in 3.34% of rabbits slaughtered in Poland [50], which indicates a favourable falling trend in the incidence of this parasitic liver damage. Rampin et al. [29], who studied pathological findings in rabbits slaughtered at slaughterhouses in Italy, found liver damage in 0.23% of all slaughtered rabbits, with hepatic coccidiosis, steatosis, and necrotising hepatitis being the most frequent causes of liver tissue damage. Conficoni et al. [27], who considered pathological lesions found during postmortem veterinary examinations of rabbits at slaughterhouses in Italy in the years 2003 to 2017, did not report liver lesions among the findings recorded at slaughterhouses at all.

A markedly lower number of liver lesions was found when species of fattening poultry were compared with end-of-lay hens. The findings also show the higher sensitivity of turkeys to the correct content of nutrients in the diet compared to other species of fattening poultry. Turkeys, in general, have a much higher protein requirement than other poultry, especially when they are young and growing. Furthermore, the amino acid composition of the diet affects the occurrence of neurodegenerative changes in the brain and liver of turkeys [51]. Pathological changes in the liver were detected in 3.38% of all slaughtered turkeys, and these were almost exclusively chronic changes. Similarly, Januskeviciene et al. [17] found that turkeys showed the largest number of pathological lesions of all studied species of poultry (broilers, turkeys, ducks). Liver lesions were the second-most frequent (45.68% of all slaughtered turkeys) after lesions on the limbs (85.79% of all slaughtered turkeys). Broiler chickens, in contrast, generally show the lowest number of pathological changes of all studied species and categories of animals [17,49], which is associated with the fact that this is also the category slaughtered at the lowest age. In agreement with the results of our study, neither Jakob et al. [30] nor Salines et al. [52] mention liver lesions as a reason for the condemnation of broiler carcasses. Buzdugan et al. [32] reported perihepatitis as the third-commonest cause of the condemnation of broiler carcasses in England. A Canadian study [31] reflects an increased condemnation rate of broiler carcasses as a consequence of pathological lesions in the liver (necrotic hepatitis, perihepatitis, and haemorrhage). Immunosuppression caused by infectious bursal disease (IBD) has been identified as the primary cause of these liver lesions.

A frequency of liver lesions only slightly higher than in broiler chickens was recorded in geese (1.09%). A probable explanation for this is the fact that this is a relatively undemanding and adaptable species that is also often reared for commercial purposes in extensive farming systems (an enclosure with pasture and access to water) that take the natural needs of this species into consideration [53]. Hepatic necrosis, which may be caused by inflammation or intoxication, is rarely detected in geese [54]. A relatively low number of liver damage was also detected in ducks (2.20%). It is, however, interesting that ducks show the highest frequency of acute changes (1.12%) of all fattening poultry, which may be caused by infection (for example, hepatitis B and reoviruses) or intoxication resulting from grazing (heavy metals, aflatoxicosis) [55,56,57]. Chronic liver damage in ducks may include steatosis or even liver cancer (caused by the hepatitis virus or aflatoxicosis) [56,58].

When comparing the incidence of chronic, acute, and parasitic changes in the liver, chronic lesions are significantly more frequent than acute or parasitic lesions in the majority of species and categories of animal species, with the exception of piglets and ducks, in which acute lesions are the most frequent, and ewes and lambs, in which parasitic lesions are the most frequent.

When the incidence of chronic lesions is compared, it is higher in adult animals than in fattening animals, and it is higher in young animals culled from the herd than in fattening animals. When individual species of adult animals are compared in terms of the occurrence of chronic lesions, they rank in the order cows, sows, ewes, and does. When individual species of fattening animals are compared they rank in the order heifers and fattening bulls, finishing pigs, lambs, and kids, and when individual species of fattening poultry and rabbits are compared they rank in the order turkeys, ducks, geese, broilers, and rabbits. 

Chronic lesions represent the dominant proportion of liver damage, and the explanations for chronic changes are similar to those for overall damage, though chronic lesions are attributed more to the long-term effects of unbalanced nutrition than to sudden changes in animal nutrition. A high production performance makes animals prone to suffering from hepatic stress, making the organ more susceptible to different challenges linked to inadequate feed management and nutritional imbalances, such as diets with an inadequate protein–energy ratio that cause the fatty liver syndrome [59]. Steatosis is a typical example of a chronic liver disease in livestock animals. The liver is the place where fat synthesis takes place, though excess fat can be stored directly in the liver under certain circumstances and this may cause health problems [10]. Severe fatty liver syndrome occurs in poultry, the development of which involves a number of factors (environmental, hormonal, genetic), some of which have not yet been fully elucidated. Laying hens and turkeys are most frequently affected by this syndrome [60].

Acute lesions correspond less to the overall incidence of liver damage than chronic lesions. Of all the species and categories of livestock animals studied, the largest number of acute changes was detected in piglets (15.97%), with a considerably smaller number found in calves (5.95%) and cows (5.23%). The largest proportion of acute lesions to the overall number of liver abnormalities was determined in piglets, broilers, and calves, i.e., in young animals in which chronic processes had not yet had time to develop fully. Acute lesions are attributed more to changes in nutrition than to the long-term action of nutritional effects. In addition to dietary causes, acute pathological processes in the liver tissue may also develop as a result of infection or intoxication. In poultry, e.g., acute lesions in the liver are found as a result of salmonellosis, campylobacteriosis, bird flu, and Marek’s disease, while intoxication is often recorded in the form of aflatoxicosis [61,62,63].

The frequency of parasitic damage is affected by species’ susceptibility to various species of parasites and the age of the animals, and these factors lead to a structure of findings different to that seen in the case of chronic and acute changes. When the number of parasitic lesions in adult animals was compared with the number in fattening animals, a significantly higher incidence was found in heifers (1.31%) compared to cows (0.69%) and fattening bulls (0.51%) and in finishing pigs (3.68%) compared to sows (0.58%), while in contrast a significantly larger number of lesions was found in ewes (7.51%) compared to lambs (3.51%) and in does (0.74%) compared to kids (0.18%). These findings correspond to the feeding of different species and categories of animals in relation to the possibilities of parasitic invasion. In cattle, heifers are, first and foremost, pasture-fed, for which reason the level of parasitic invasion in heifers is higher than that seen in cows and fattening bulls, which are fed in stalls with feed mixtures, silages, and haylage. Pig nutrition is based on feed mixtures, though in view of the danger of invasion by roundworms, better parasite hygiene care on farms is devoted to sows due to their value and reproductive benefit than to finishing pigs, in which parasitic damage to the liver (ascariasis) is recorded most often (3.68%) of all categories of pigs. Infestation of pig farms with roundworms was also mentioned by Ceccarelli et al. [45], who reported parasitic hepatitis as the cause of condemnation in 96.2% of all condemned pig livers.

In sheep, grazing on pasture leads to parasitic invasion, the impact of which is higher in ewes, in which it is more frequent and longer-lasting than it is in lambs in view of their age. Sheep (ewes and lambs) are the only species in which liver lesions of parasitic origin predominated. This finding is also confirmed by Ceccarelli et al. [45], who found that as many as 96.2% of all liver lesions found in sheep were of parasitic origin. Torina et al. [46] draw attention to the fact that as much as 80% of pathology in sheep and goats is associated with the presence of parasites. Besides pasturing, differences in the antibody isotype responses of sheep and cattle to fasciolosis may play an important role in the frequency of liver lesions detected. Phiri et al. [64] documented a difference in the IgG2 isotype response in cattle (resistant) compared to sheep (susceptible).

Does are reared both in stalls and on pasture, for which reason the frequency of parasitic lesions is low, though higher than in kids in view of the longer period of possible infection in adult animals. In contrast to our study, Ceccarelli et al. [45] recorded a significant quantity of pathological changes of parasitic origin in the liver (primarily ascariasis and distomatosis) in practically all livestock species studied.

When the number of parasitic lesions in young culled from the herd was compared with that in fattening animals, a significantly higher incidence was found in heifers (1.31%) and fattening bulls (0.51%) compared to calves (0.13%). A higher incidence was also found in finishing pigs (3.68%) compared to piglets (1.21%). The impact of parasitic invasion is likely higher in heifers and fattening bulls than in calves due to their older age and, therefore, the longer period of time for possible parasitic invasion than in calves. The same is also true when finishing pigs and piglets are compared.

When adult animals of individual species are compared, the number of parasitic lesions is significantly higher in ewes (7.51%) than in does (0.74%), cows (0.69%), and sows (0.58%). This ranking of parasitic lesions in adult animals by species likely corresponds to the method of feeding associated with the possibility of parasitic invasion, with the number being high in ewes, which graze over the long term in the open with a large possibility of parasitic infection. Cows and sows are primarily housed in stalls, while does are reared both in stalls and on the pasture, with limited possibilities for parasitic invasion, for which reason there are significantly fewer parasitic lesions found in the liver in these species compared with ewes.

When fattening animals are compared by individual species, a significantly higher incidence of parasitic lesions is found in finishing pigs (3.68%) and lambs (3.51%) in comparison with heifers (1.31%), fattening bulls (0.51%), and kids (0.18%). This ranking of parasitic damage in fattening animals by species corresponds to the method of feeding associated with the possibilities of parasitic invasion, and is higher in lambs, which graze in the open with ewes and are exposed to considerable opportunities for parasitic infection. Heifers are also often kept on pasture, though considering the nature of these pastures in the Czech Republic (generally not pastures in damp and waterlogged areas) they are characterized by lower opportunities for parasitic invasion. The occurrence of parasitic invasion with roundworms is found in finishing pigs and this is treated only to an extremely limited extent in view of the period of fattening and protection of the meat against residual antiparasitics, for which reason the incidence of parasitic lesions in the liver of finishing pigs is recorded more often. Kids are generally fed in stalls rather than by grazing in view of the period of their fattening, for which reason the possibilities for parasitic invasion are limited. Parasitic lesions in rabbits are extremely rare in view of their feeding with granules. They may occur in sporadic cases in connection with hepatic coccidiosis in animals kept on small farms where feed is also provided in the form of grass and hay. Parasitic findings are rare in poultry.

## 5. Conclusions

Varying proportions of animals suffering from liver lesions were found in different animal species and categories slaughtered for human consumption at the slaughterhouse. The results form a body of knowledge for measures to improve the health and liver condition of food animals. Besides the species-specific differences, the effect of age, rearing method, housing system, and nutrition should be considered when adopting measures aimed to improve the liver health of farm animals. Such measures should be adopted namely in calves, piglets, and turkeys, in which the incidence of liver damage showed a significantly increasing trend during the monitored period.

## Figures and Tables

**Table 1:** Numbers of slaughtered animals and the occurrence of liver damage broken down into acute, chronic, parasitic, and other damage for individual categories of slaughtered animals.

**Origin of Damage**	**Species/Category of Animals** **(Number of Slaughtered Animals)**										
	Cows (1,348,393)	Heifers (315,406)	Fattening bulls (1,214,298)	Calves (120,238)	Sows (683,912)	Finishing pigs (29,628,524)	Piglets (152,088)	Ewes (26,026)	Lambs (132,553)	Does (1680)	Kids (7690)
	**Occurrence of Liver Damage (%)**										
Acute	5.23 ^b^	1.35 ^b^	0.40 ^c^	5.95 ^b^	1.00 ^b^	0.22 ^c^	15.97 ^a^	0.09 ^c^	0.04 ^c^	0.00 ^c^	0.00 ^c^
Chronic	39.13 ^a^	11.41 ^a^	7.04 ^a^	11.49 ^a^	15.93 ^a^	7.35 ^a^	15.21 ^b^	5.35 ^b^	1.18 ^b^	3.42 ^a^	0.41 ^a^
Parasitic	0.69 ^d^	1.31 ^b^	0.51 ^b^	0.13 ^c^	0.58 ^c^	3.68 ^b^	1.21 ^c^	7.51 ^a^	3.51 ^a^	0.74 ^b^	0.18 ^b^
Other	1.33 ^c^	0.10 ^c^	0.01 ^d^	0.10 ^d^	0.01 ^d^	0.00 ^d^	0.00 ^d^	0.02 ^d^	0.00 ^d^	0.09 ^c^	0.00 ^c^
**Origin of Damage**	**Species/Category of Animals** **(Number of Slaughtered Animals)**										
	Rabbits (2,403,779)		End-of-lay hens (23,116,811)		Broiler chickens (1,328,693,065)		Turkeys (1,516,365)		Geese (52,360)		Ducks (36,296,955)
	**Occurrence of Liver Damage (%)**										
Acute	0.00 ^c^		2.55 ^b^		0.03 ^b^		0.00 ^d^		0.00 ^c^		1.12 ^a^
Chronic	0.04 ^a^		17.76 ^a^		0.04 ^a^		2.75 ^a^		1.05 ^a^		1.08 ^b^
Parasitic	0.00 ^b^		0.00 ^d^		0.00 ^d^		0.01 ^c^		0.03 ^b^		0.00 ^c^
Other	0.00 ^c^		0.01 ^c^		0.00 ^c^		0.62 ^b^		0.00 ^c^		0.00 ^d^

^a–d^ values in columns within the same species/category of animals with different letters of superscripts are significantly different at *p* ˂ 0.05.

**Table 2:** Spearman rank correlation coefficients of the 12-year data within the animal category.

Species/Category of Animals	Correlation Coefficient	Significance (*p*)	Species/Category of Animals	Correlation Coefficient	Significance (*p*)
Cows	−0.4825	0.1121	Does	0.1399	0.6646
Heifers	−0.8462	0.0005	Kids	−0.7042	0.0106
Fattening bulls	−0.6923	0.0126	Rabbits	0.6643	0.0185
Calves	0.8951	0.0001	End-of-lay hens	−0.9021	0.0001
Sows	−0.0350	0.9141	Broiler chickens	−0.2657	0.4038
Finishing pigs	0.0699	0.8290	Turkeys	0.6014	0.0386
Piglets	0.9161	0.0000	Geese	0.2867	0.3663
Ewes	−0.7552	0.0045	Ducks	0.3986	0.1993
Lambs	−0.7902	0.0022			

## Data Availability

Data for analysis were obtained from the information system of the State Veterinary Administration of the Czech Republic. The datasets generated and analyzed during the current study are available from the corresponding author on reasonable request.
